# *De Novo* ORFs in Drosophila Are Important to Organismal Fitness and Evolved Rapidly from Previously Non-coding Sequences

**DOI:** 10.1371/journal.pgen.1003860

**Published:** 2013-10-17

**Authors:** Josephine A. Reinhardt, Betty M. Wanjiru, Alicia T. Brant, Perot Saelao, David J. Begun, Corbin D. Jones

**Affiliations:** 1 Department of Biology, University of North Carolina at Chapel Hill, Chapel Hill, North Carolina, United States of America; 2 Department of Biology, University of Maryland at College Park, College Park, Maryland, United States of America; 3 Center for Population Biology, University of California, Davis, Davis, California, United States of America; University of Texas at Arlington, United States of America

## Abstract

How non-coding DNA gives rise to new protein-coding genes (*de novo* genes) is not well understood. Recent work has revealed the origins and functions of a few *de novo* genes, but common principles governing the evolution or biological roles of these genes are unknown. To better define these principles, we performed a parallel analysis of the evolution and function of six putatively protein-coding *de novo* genes described in *Drosophila melanogaster*. Reconstruction of the transcriptional history of *de novo* genes shows that two *de novo* genes emerged from novel long non-coding RNAs that arose at least 5 MY prior to evolution of an open reading frame. In contrast, four other *de novo* genes evolved a translated open reading frame and transcription within the same evolutionary interval suggesting that nascent open reading frames (proto-ORFs), while not required, can contribute to the emergence of a new *de novo* gene. However, none of the genes arose from proto-ORFs that existed long before expression evolved. Sequence and structural evolution of *de novo* genes was rapid compared to nearby genes and the structural complexity of *de novo* genes steadily increases over evolutionary time. Despite the fact that these genes are transcribed at a higher level in males than females, and are most strongly expressed in testes, RNAi experiments show that most of these genes are essential in both sexes during metamorphosis. This lethality suggests that protein coding *de novo* genes in Drosophila quickly become functionally important.

## Author Summary

*De novo* genes are protein-coding genes with no clear homology to previously existing protein-coding genes. Since their discovery in Drosophila and other species including humans, their existence has been controversial, with some doubt as to how they would arise, whether they produce proteins, and whether they could possibly perform any useful function. Here, we show that RNAi of several Drosophila *de novo* genes causes lethality – in fact, a higher proportion of *de novo* genes cause lethality than was found in a similar screen of other young and novel genes. Further, we find that *de novo* genes do produce proteins in the majority of cases and that in some cases, they were transcribed prior to the emergence of an open reading frame. Our data suggests that Drosophila *de novo* genes are an unexpected avenue for non-coding DNA sequences to contribute evolutionary and functional novelty.

## Introduction

Most new genes arise from the duplication or rearrangement - in whole or in part - of existing genes [1], [2]. These new genes are typically structurally and functionally similar to their progenitors. In contrast, protein-coding genes may also evolve *de novo* from previously non-coding sequences, making them lineage-specific and unlike any existing protein. *De novo* genes were once thought to be vanishingly rare, or even impossible [3]. Subsequent work suggests instead that these brand-new genes may make up a significant proportion of novel genes and that some have important functions.

The first experimental evidence of *de novo* genes in Drosophila came from studies identifying a handful of protein-coding genes apparently specific to the *D. melanogaster* [4] and *D. yakuba* [5], [6] lineages respectively. Analysis of multiple genomes in Drosophila had previously indicated that intergenic DNA contained abundant protein-coding potential [7], but many strongly predicted genes were not functional [8]. The early *de novo* gene papers identified proteins that were lineage-specific and were also were stably expressed in a specific tissue (the testis). Because most functional genes were believed at that time to produce proteins, these early efforts focused on the *de novo* emergence of proteins from regions lacking that ORF ancestrally. Genes that had high similarity hits in close relatives were excluded, though conservation of synteny was required [4]. This prevented mischaracterizing novel genes that arose through some other mechanism - such as duplications of functional exons - as *de novo* evolved. A similar strategy was later used to identify *de novo* protein coding genes in yeast [9] and mammals [10]. In contrast to Drosophila, work focused on humans identified genes with *high* similarity matches in the comparison species coupled with a lineage-specific loss of a mutation disabling the open reading frame (e.g. *de novo* proteins) [11], [12]. Regardless of the detection strategy used, the early work focused on the evolution of a novel protein from DNA sequence thought to be non-coding, and the evolution of lineage-specific transcription was largely ignored. As the increasing importance of non-coding RNA genes became broadly recognized, efforts to identify *de novo* evolution of non-coding RNA genes began. Heinen and colleagues [13] identified a case of novel transcription from a previously untranscribed region in mice. This novel transcript did contain an ORF, but the researchers argued that the short peptide encoded was unlikely to be functional. More recently, some human *de novo* proteins were found to have likely arisen from previously transcribed non-coding RNA sequences [12], implying that the evolution of a *de novo* protein may occur either before or after transcription of a previously non-coding region begins.

**Figure 1:**
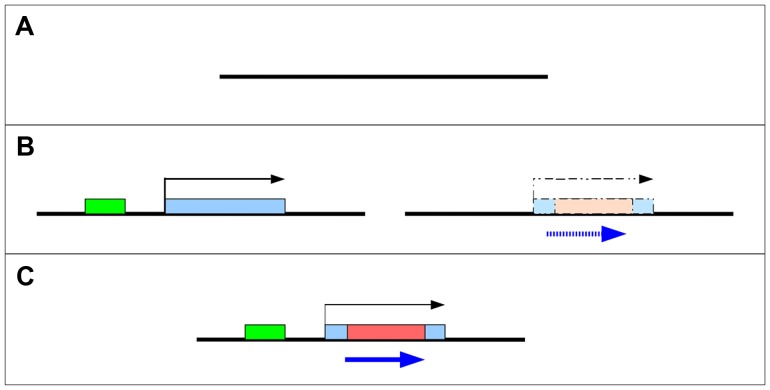
Two models for the origin of *de novo* genes. *De novo* genes may emerge and evolve into protein coding genes (C) from non-coding sequences (A) through one of several intermediate steps (B). Left - a novel non-coding RNA becomes transcribed after a new promoter (green) is recruited. Right - a “cryptic” ORF (blue) is present prior to the origin of transcription.

What is clear is that for a protein-coding gene to arise *de novo* it must evolve both transcriptional and protein-coding potential. In principle, these events could occur in either order (Figure 1B). If a new open reading frame (ORF) evolves within a transcribed region (such as a non-coding RNA), it is more likely to ultimately be translated than an ORF that evolves in a region of untranscribed DNA (Figure 1B left). Alternatively, an ORF may exist in the ancestral state, but not be expressed until transcription is initiated through acquisition of regulatory machinery (Figure 1B right). In either case, ORFs may subsequently expand through loss of stop codons and/or exon gain. These models are not mutually exclusive and intermediate models have been proposed – for example, occasional read-through transcription of genes [4], translation of small ORFs from non-coding RNA, or other partial gene states are expected to occur commonly. Indeed, both Yeast [14] and Drosophila [15] contain hundreds of these “proto-genes” which may subsequently evolve into *de novo* protein coding genes.

Despite the wide array of studies identifying *de novo* genes using multiple approaches in many taxa, the number of genes with functional characterization remains small. A recently identified yeast *de novo* gene, BSC4, is important for DNA repair [9], [16]. The *Drosophila melanogaster de novo* genes, *CG31406* [17] and *CG31909* [18] both showed pupal lethality in large RNAi screens and the mouse *de novo* gene *Pldi* affects male fertility [13]. The analysis of *de novo* gene function in humans has been restricted to analysis of previously existing gene expression and association with disease phenotypes in GWAS data, but are suggestive of function in the brain for one gene [19]. Here we combine an analysis of the evolutionary history – including analysis of sequence evolution and expression – with functional studies of six *D. melanogaster de novo* genes previously reported in the literature [4], [20]. These six *de novo* genes represent a variety of “steps” in the evolution of *de novo* genes, consistent with previously described gradual models of *de novo* gene evolution [4], [9], [14]. Some *de novo* genes are specific only to *D. melanogaster*, *D. simulans*, and *D. sechellia*. Others have a deeper evolutionary history, with evidence of the evolution of transcription (but not necessarily an ORF) occurring in the common ancestor of *D. melanogaster* and *D. yakuba*/*D. erecta* or earlier. We find that two of the genes were clearly transcribed prior to the evolution of an open reading frame, supporting the concept that *de novo* proteins may evolve from non-coding RNA genes. In four other cases, an open reading frame and transcription appear to have co-occurred in the same evolutionary interval. Knockdown of *de novo* genes with RNAi showed that these *de novo* genes are important to organismal fitness. Finally, our data show that despite arising through different mechanisms, *D. melanogaster de novo* genes share evolutionary and functional similarities.

## Results

### Ages and evolutionary trajectory of *de novo* genes vary

We investigated *de novo* genes previously described [4], [20] as having arisen recently in the *D. melanogaster* lineage (both *D. melanogaster* subgroup and *D. melanogaster* specific) – along with other internal candidates (Methods) – and reassessed whether they qualify as *de novo* protein-coding genes using current genomic resources. For each gene, we determined whether proteins had arisen recently from apparently non-coding DNA by tBLASTn of the protein-coding regions to all 12 Drosophila genomes, as well as comparing to UCSC's BLASTZ alignments from *D. yakuba, D. erecta, D. ananassae, D. simulans, and D. sechellia*). This eliminated a number of candidates from consideration either because they were collinear to highly diverged putative protein-coding sequences in species previously analysed, or because one of the species in the 12 genomes that was not previously analyzed contained a potential ortholog (see Table S1 for the full list of candidates).

**Figure 2:**
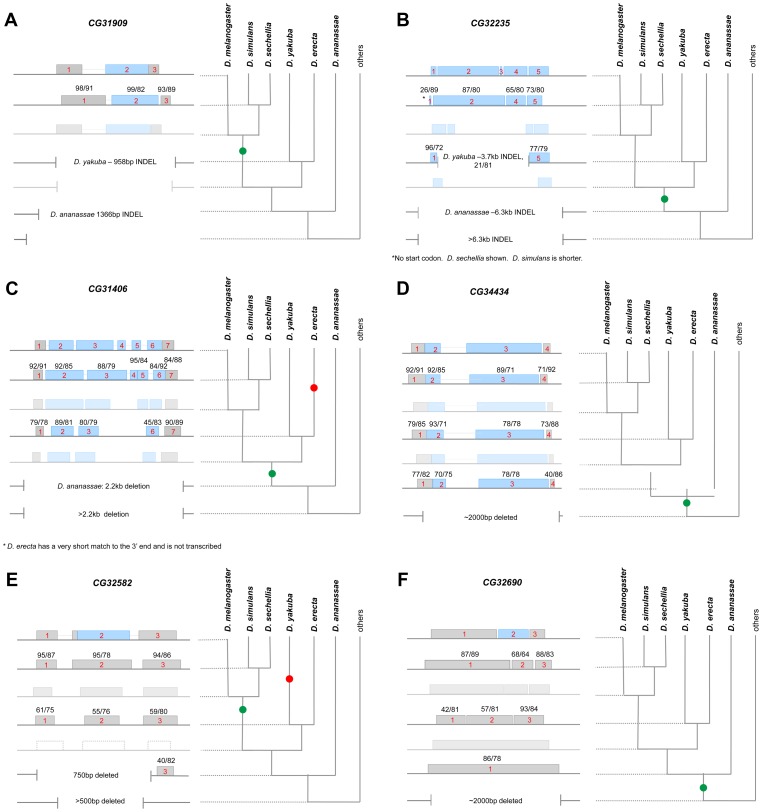
Stepwise gene model evolution of six *D. melanogaster de novo* genes. We used BLASTZ alignments as well as our own MAUVE alignments to infer the evolution of six *D. melanogaster de novo* genes – *CG31909* (A), *CG33235* (B), *CG31406* (C), *CG34434* (D), *CG32582* (E) and *CG32690* (F). The current *D. melanogaster* gene model is shown on top, and blocks of sequence that are collinear and align to parts of the *D. melanogaster* gene (by BLASTZ) are shown below. Blue blocks represent putative protein coding sequence, grey blocks non-coding sequence. *D. simulans*, *D. yakuba*, and *D. ananassae* collinear blocks are shown as appropriate, with the size of the block indicating the relative length of the alignment. The proportion of *D. melanogaster* bases aligned and the sequence similarity of aligned bases are shown on each block (proportion/similarity). Large scale deletions are shown using vertical lines. The inferred gene model at the nodes is also shown as faded blocks. Finally, expression was measured (using RT-PCR) in each species where collinear sequence could be found. Species where expression was detected are bolded on the phylogeny and the green dot on the phylogeny indicates the inferred start of transcription. A red dot indicates cases where transcription was lost or the gene was lost in that lineage as described.

For the remaining six genes, we extracted the UCSC BLASTZ alignments for sections of each gene (5′UTR, all CDS exons, and 3′UTR), then used the pairwise sequence alignment program water to calculate the sequence identity and the proportion of the *D. melganogaster* sequence conserved between *D. melanogaster* and each of the other species in the alignment (Figure 2). We found that five of the six genes could be aligned to fragments of sequence from species as far diverged as *D. yakuba* or *D. erecta*, and in the case of *CG34434*, *CG31406*, and *CG32235*, sequences that overlapped with the *D. melanogaster* open reading frame in these species were not interrupted by stop codons indicating that if transcribed and translated, a highly diverged protein or peptide may be produced in these closely related species. In addition, sequences collinear to portions of the *CG34434* CDS and part of the *CG32690* UTR could be found in *D. ananassae* (Figure 2D and 2F). These sequences are highly diverged and major changes in size and structure were apparent in many cases.

*CG32582* and *CG32690* can be distinguished from the other *de novo* genes because they appear to have an open reading frame that is unique to *D. melanogaster* alone. Collinear sequences in *D. simulans* and other species carry disabling mutations that greatly truncate any potential ORF (Figure 2, Supporting data). *CG31909* is well-conserved in *D. simulans* and *D. sechellia* but no sequences similar to the CDS can be found in any other species. Interestingly, while the *CG31909* CDS is novel, the 5′ UTR of *CG31909* contains similarity to a short transposable element – perhaps sequence from elsewhere in the genome was inserted in the ancestor of *D. simulans* and *D. melanogaster* through movement of that transposable element. The lack of sequence similarity of the CDS for any sequence in any genome other than *D. melanogaster* and its two sister species makes it difficult to determine the origin of this sequence. *CG31909* also has a near exact paralog (98% amino acid identity) in *D. melanogaster* (now annotated as *CG43800* as of Flybase r5.45) that is specific to *D. melanogaster*. Interestingly, an RNAi screen of Notch signaling genes showed RNAi of *CG31909* to be semi lethal [18]. The remaining genes (*CG31406*, *CG33235*, and *CG34434*) have undergone structural changes after their origins resulting in increases over time in the size of the total gene (*CG31406* and *CG33235*) the size of the CDS (all three), and the number of exons (*CG31406*) (Figure 2).

### *De novo* genes became expressed through a variety of mechanisms

*De novo* protein-coding genes might evolve from previously non-coding but transcribed sequences (“Transcription first” model, Figure 1). Alternatively, a previously untranscribed ORF could arise through random mutation, and only later become transcribed (“Proto-ORF” model, Figure 1). Of course, these models are not mutually exclusive, and do not rule out other intermediate possibilities – such as transient transcription of an existing ORF later becoming stably transcribed (see [6], [9], [14]). As described above, in all cases these sequences were highly diverged at both the sequence and structural level (Figure 2). We used qRT-PCR to measure transcription of these genes in species where collinear sequences could be found, regardless of protein-coding potential (Figure 2, with bolded text indicating species where transcription could be detected). With the exception of two genes, we were able to detect expression of transcripts in all species in which collinear sequence could be clearly identified (*CG31406* was expressed in *D. yakuba* but not *D. erecta* despite alignable sequence being present in both species; *CG32582* was not expressed in either *D. erecta* or *D. ananassae*). These data suggest that the *de novo* evolution of expression can predate the evolution of the ORF and that existence of a proto-ORF was not a prerequisite for the evolution of transcription of the *de novo* gene.

In the cases where an ORF was present (*CG31909*, *CG34434*, *CG31406*, and *CG33235*), we surmise that the origin of the ORF and the evolution of stable transcription arose at around the same time. While these data are consistent with the hypothesis that transcription arose from nascent ORFs in the genome (proto-ORF model), we cannot conclude that the proto-ORF existed first—transcription may have evolved first and then an ORF shortly thereafter. On the other hand, in cases where the sequence was clearly non-coding and stably transcribed prior to the evolution of an ORF (*CG32690* and *CG32582*), we can conclude that the transcription-first model applies.

We next mined the EBI PRIDE proteomic database for evidence that the extant ORFs were translated. Four of the six *de novo* genes – all but the newest ORFs, *CG32582* and *CG32690* –expressed peptides in early embryos ([21]–[24], Table S2). It is unknown if the short proto-ORFs of these four genes are being translated in other species or if the other two genes are translated in other, less deeply surveyed tissues in *D. melanogaster*. All six genes have sequence features consistent with post-translational cellular localization - *CG32690*, *CG32582*, and *CG34434* have secretory signals, whereas *CG31909* has a nuclear signal and *CG33235* is predicted to be localized to the mitochondria. In sum, we have evidence for translation of the ORF in all four of the “proto-ORFs”, but not for the two “transcription-first” genes. These data do not rule out the possibility that *CG32690* or *CG32582* are translated in *D. melanogaster* as only one tissue (embryos) was deeply surveyed, but these data are consistent with the interpretation that genes arising through a transcription-first mechanism are less likely to produce peptides and that their biological activity is tied to the evolution of a novel RNA, rather than a novel protein.

### Testes biased expression is conserved across species

**Figure 3:**
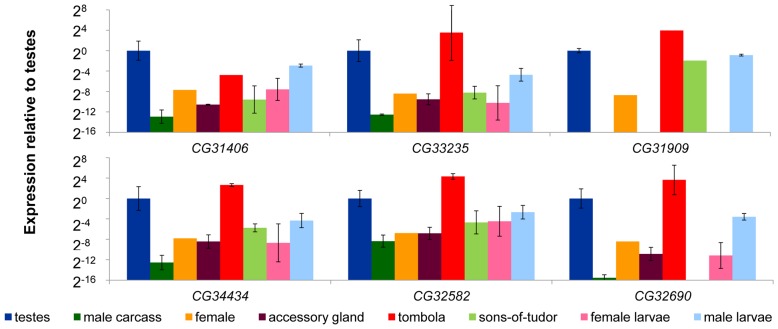
*De novo* genes exhibit male-biased and germline-dependent expression. We compared the expression of six *D. melanogaster de novo* genes (*CG31406*, *CG33235*, *CG31909*, *CG34434*, *CG32582*, and *CG32690*) in a variety of tissues dissected from *D. melanogaster* using qRT-PCR. Expression of each gene was measured using Actin as a reference (similar results were obtained using GPDH as the control gene, data not shown). Expression results are shown relative to the testes sample, and was highest in the testes (testes and tombola columns were both testes samples), and was reduced in testes of males lacking a gremline (*sons-of-tudor*, light green). In the case of *CG31406*, expression was reduced in flies carrying a meiotic arrest mutation (*tombola*, red), suggesting it may be functioning in the post-meiotic germline. Finally, we found that male larvae express all six genes at a higher level than female larvae (pink compared to light blue).

Prior work shows that *de novo* genes in Drosophila tend to exhibit male-biased expression [4], and are expressed at their highest levels in L3 larvae, pupae, adult males, and the adult reproductive system [25]. We compared expression in *D. melanogaster* in adult testes, male accessory glands, the remainder of the male tissues, and adult females. In addition, we sexed L3 larvae [26] and measured expression in male and female larvae. We found male-biased expression in all six genes. Expression of *de novo* genes was at its highest in the testes, and male larvae expressed at a higher level than female larvae (Figure 3). We also found that lack of a male germline (Figure 3 *sons-of-tudor*, light green) reduces but does not typically eliminate expression (transcription of *CG32690* was undetectable in the *sons-of-tudor* testes). This suggests that these *de novo* genes are contributing to the development and maturation of sperm, but likely perform other functions as well. Following on this result, we determined whether these genes were regulated downstream of a spermatogenesis specific gene by measuring expression in a *tombola (tomb)* mutant background. *tombola* is a transcription factor known to activate expression of a suite of genes important during male meiosis in Drosophila [27]. We found that expression of *CG31406* was reduced in the *tomb* mutant background (Figure 3, red) implying expression of this gene is partially dependent on an intact meiotic arrest pathway. The other genes, however, did not appear to be affected by *tomb*, suggesting that though they are expressed at a high level in the male germline they either operate up-stream of *tomb* or are regulated by a parallel pathway.

**Figure 4:**
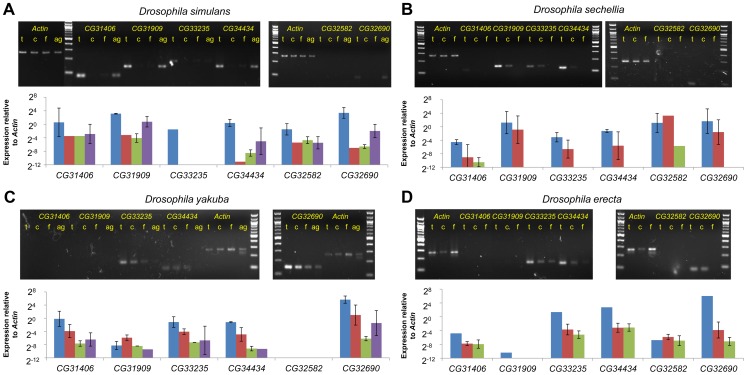
Testes biased expression of *de novo* genes is conserved across species. We compared the expression of sequences or genes that were collinear to *D. melanogaster de novo* genes across a number of tissues in the five species of the *melanogaster* subgroup. In *D. sechellia* (B) and *D. erecta*, (D) we dissected male reproductive tracts from flies, and compared expression across the reproductive tracts (Testes “t”, blue), the remainder of the male (Carcass “c”, red), and whole females (Females “f”, green). In *D. yakuba* (C) and *D. simulans*, (A) we further dissected male reproductive tracts into testes and accessory glands (“ag”, purple). When available, two biological replicates are shown. Expression shown is relative to the same set of *Actin5c* primers across all 5 species. In those cases where the gene was expressed at a moderate level in any tissue (shown with a *), expression was always higher in the testes than in female-derived tissues suggesting preservation of testes-bias in expression. For *CG31909*, which is almost entirely deleted in *D. yakuba* and *D. erecta*, primers were designed to the closest alignable sequence to the *D. melanogaster* gene region, and expression was not detected. *CG32582* was deleted in *D. yakuba* and expression was not detected in *D, erecta*. Despite not containing an open reading frame in *D. simulans* and *D. sechellia*, however, *CG32582* was expressed in a testes-biased manner in these species. Likewise, *CG32690* was expressed stably in both *D. yakuba* and *D. erecta* despite the presence of no ORF in these species. Finally, although the band is not visible for CG31406 in *D. yakuba* on this gel, the ct values for the testes samples (but not other samples) indicated expression similar to the *D. simulans* testes samples.

Next, we compared expression levels of collinear expressed sequences in tissues (testes, male carcass, and female) from *D. simulans*, *D. sechellia*, *D. yakuba* and *D. erecta* (Figure 4A–D). Despite radical structural and sequence changes, testes-biased expression of all *de novo* genes was conserved for species in which expression could be readily detected. It has been suggested that *de novo* genes might occasionally be transcribed spuriously (possibly due to a permissive transcriptional environment [28]) prior to recruitment of a more specific promoter upon evolution of a novel function. This idea predicts that expression levels should vary stochastically across species. Our results suggest instead that *de novo* genes have been expressed in a biased manner from the moment transcription originated. Additionally the *sons-of-tudor* and *tomb* data suggests that active regulation of these genes' expression evolved early.

### RNAi of *D. melanogaster de novo* genes affects viability and male fertility

**Figure 5:**
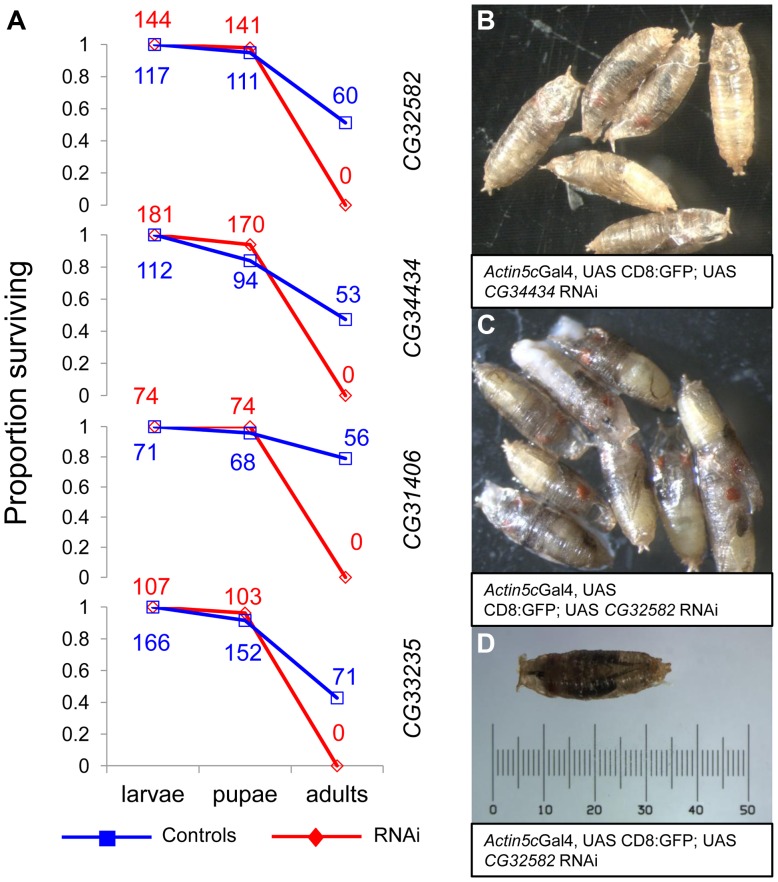
RNAi of four *D. melanogaster de novo* genes causes arrest at the pharate stage. We knocked down expression of four *de novo* genes using phiC31 UAS-RNAi lines (see methods, Figure S1) and found that adult RNAi flies did not eclose. (A) By using a GFP marked *Actin*-Gal4 driver, we found that RNAi (red, diamond) and control (blue, square) flies had similar death rates before the adult stage (wandering larvae were sorted for GFP status and subsequently allowed to develop in separate vials). At the time of pupation, survival rates were not significantly different, but prior to the time of eclosion all RNAi individuals had died (A). By observing developing pupae each day, we found that RNAi pupae but not control pupae arrested at the pharate adult stage, just prior to eclosion (*CG34434* (B) and *CG32582* (C) are shown, other crosses similar) with a number of fully pigmented adult features visible (e.g., eyes, wings, legs). A single *CG32582*-RNAi pupa is shown with a scale for reference (D). The raw number of animals of each genotype are shown as numbers on the plot. As observed with the *Actin*-Gal4 cross, control but not RNAi adults were produced for all of the crosses.

The consistency of testes-biased expression of the genes across species led us to hypothesize that these genes may function primarily as male fertility genes. Contrary to our expectation, we found that RNAi knockdown of the four *de novo* genes we were able to assay strongly affected viability. RNAi stocks from the VDRC's [29] phiC31 library (also known as “KK stocks”) crossed with a ubiquitous *Actin5C*-GAL4 driver (y^1^ w*; P{Act5C-GAL4}25FO1/CyO, y^+^), produced no RNAi offspring for the four genes assayed (*CG31406*, *CG32582*, *CG34434*, *CG33235*), We further characterized the viability phenotype using a driver line that included a GFP marker (y^1^ w*; P{Act5C-GAL4}25FO1, UAS:CD8:GFP/CyO, y, donated by S. Chen) and found that lethality occurred in all four cases at the late pharate adult stage, just prior to eclosion (Figure 5). Our observation of pharate-stage lethality is consistent with previous work showing RNAi of *CG31406* leads to pharate-stage death [17]. This result suggests that these four *de novo* genes may be essential. To rule out spurious effects of RNAi, we crossed all RNAi lines to an additional ubiquitious *Tubulin*-GAL4 driver (y^1^ w^*^; P{tubP-GAL4}LL7/TM3, Sb^1^, Bloomington #5138) as well as a driver that targeted testes and various essential larval tissues (larval fat body, gut, leg discs, and salivary glands, w^1118^; P{GawB}c564, Bloomington #6982) with the same result. We also drove RNAi expression of a negative control phiC31 RNAi stock (*Gr22c*) using the *Actin5c*GAL4 driver and saw no lethality, as expected. Finally, we measured the extent of RNAi knockdown for all lines and found that RNAi samples had weaker expression of the target gene than controls (Figure S1B), whereas there was no significant knockdown of genes predicted to be potential off-targets by sequence similarity (Figure S1C), which is consistent with other studies using these lines that show that off-target effects are rare [18].

We also obtained P-element RNAi lines from the VDRC (also known as “GD stocks”) for four of the six genes (*CG33235*, *CG31406*, *CG31909*, and *CG34434*). Due to their random placement in the genome, the P-element library produce more variable knockdown than the “KK” stocks in which the construct is placed in the well characterized phiC31 site (expression of the “GD” stock was weaker for two of the three genes for which we had both a “KK” and a “GD” stock, Figure S1). Using the same design as above, all “GD” lines produced viable progeny of both sexes. We confirmed partial knockdown (Figure S1A) of the target genes in adults from three of the crosses (*P*<0.05), but *CG31909* did not show knockdown (*P* = 0.42). This gene showed partial pupal lethality in an earlier study where its expression was driven by *pannier* promoter [18], suggesting our ubiquitious driver did not express RNAi strongly enough to knock down expression. *CG34434* GD-RNAi showed robust (∼40-fold) knockdown and a semi-lethal phenotype in adults (Table 1), with males more affected than females, whereas *CG31406*, *CG31909* and *CG33235* GD RNAi had no significant affect on overall viability. In addition, *CG34434* GD-RNAi males had a dramatically reduced lifespan compared to control males (Figure S2A). Although overall viability was not affected in the other three genes tested, female-biased skews in the sex-ratio of F_1_ adults were observed for three of the four genes tested (compared to the expected 50∶50 sex-ratio and the observed sex-ratio of controls). As parents do not carry RNAi - only offspring - the skewed sex-ratios cannot be the result of sex-chromosome meiotic drive. Indeed, we saw no bias in sex ratio of F2 offspring in subsequent experiments (described below). Instead, these findings could be the result of a male viability defect of the same type that caused complete lethality in the KK-RNAi lines, or in principle, increased viability among RNAi females.

**Table 1:** Effects of RNAi using “GD” lines targeting *de novo* genes.

	RNAi males	Control males	RNAi females	Control females	RNAi/control	Viability P-value (Fisher's test)	RNAi sex ratio*	Control sex ratio*	Sex ratio P-value (bionomial test)
CG31406-GD	77	58	87	77	1.21	0.5595	0.4695	0.4296	0.86665
CG31909-GD	42	35	64	37	1.47	0.28	0.3962	0.4861	0.00039
CG33235-GD	80	82	112	78	1.20	0.0868	0.4167	0.5125	0.00483
CG34434-GD Trial 1	22	81	46	95	0.39	0.065	0.3235	0.4602	0.01533
CG34434-GD Trial 2	193	559	355	599	0.47	<0.0001	0.3522	0.4827	<0.0001

* Males/total offspring.

**Figure 6:**
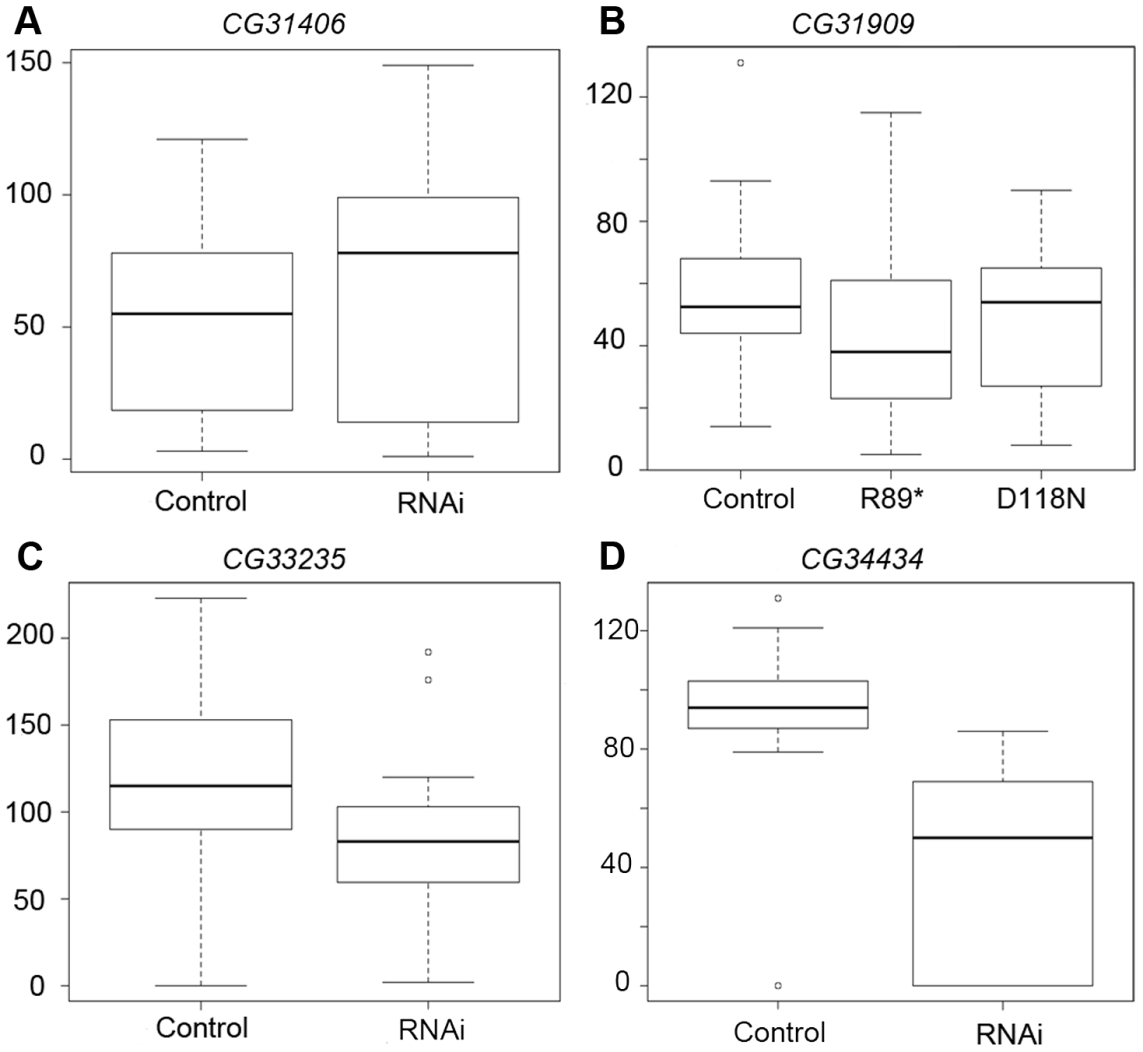
RNAi of *CG34434* leads to a reduction in male fecundity. We measured fecundity in male flies by mating F1 RNAi, mutant or control males to 1–2 females overnight, and then counting the number of offspring produced per female over a 10-day period. For *CG31406* (A), *CG33235* (C), and *CG34434* (D) we compared fecundity between the control (curly) and RNAi (straight winged) F1 males produced by crossing the “GD” UAS-RNAi stocks to an Actin-Gal4 driver (see methods). As *CG31909*-RNAi did not produce knockdown of the target gene (Figure S1), we generated a series of Fly-TILL mutants for this line, and crossed a premature termination codon mutant (R89*) and a nonsynonymous mutant (D118N) to a deficiency covering the gene (w*^1118^*; Df(2L)BSC291/CyO). We crossed w^1118^ to the same deficiency as a control, then compared the Fly-TILL mutants to the w^1118^ control using the same single day mating assay (B). Only *CG34434*-RNAi males (D) showed a significant decrease in fecundity compared to their control siblings (p<0.0001). For *CG31909*, *CG33235*, and *CG34434*, the data shown are the results of the first day of a 5-day long sperm exhaustion assay whereas for *CG31406* only the 1-day single fly matings trials were attempted.

Using males from the three RNAi crosses that produced viable males, we proceeded to measure effects on male fertility and sperm production using two assays (Figure 6, Figure S2). We mated single RNAi and control F1 males to *w^1118^* females, and found that total fertility was reduced by RNAi of *CG34434* (Figure 6D, Student's t-test *P*<0.0001) but not *CG31406* (Figure 6A) or *CG33235* (Figure 6C). We extended these findings using a sperm exhaustion assay [30] for two of the genes (*CG33235* and *CG34434*). Sperm exhaustion measures the ability of a male to continue to produce viable progeny when challenged with multiple females over a five day period and can be more sensitive to subtle differences in fertility. *CG34434* GD-RNAi males performed even more poorly during the later days of the assay than in the single-day mating experiments, but there was still no effect on the fecundity of *CG33235* GD-RNAi males using this assay (Figure S2B). Rather than having a direct effect on fertility, we suspect that *CG34434* GD-RNAi males are weaker overall as indicated by their shortened lifespan (Figure S2A) and hence were less able to mate successfully. That said, RNAi of these genes using a more specific and powerful male germline driver might reveal specific defects in spermatogenesis or fertility that we were unable to detect in this preliminary analysis.

Because we were unable to knock down expression of *CG31909* using RNAi, we produced TILLing lines for *CG31909* [31] obtaining an allele with a premature termination codon (PTC, predicted to truncate 40% of the protein) as well as a number of nonsynonymous mutations. We crossed the PTC line (SH2_0024:R89*) to a deficiency covering the *CG31909* gene region (*w^1118^*; Df(2L)BSC291/CyO, Bloomington #23676) and the PTC allele did not alter expression (data not shown), which was not unexpected as nonsense mediated decay in Drosophila does not typically affect expression if PTCs occur within ∼400 bp of the polyA signal [32], [33]. None of the alleles appeared to affect viability. We used the same two fecundity assays described above to determine whether the PTC a protein-coding mutation (D118>N) reduced fertility and saw no effect of the flyTILL lines on performance compared to controls (a D->N mutation at position 118 and *w^1118^* crossed to the same deficiency, Figure 6B, Figure S2B). This could be for a number of reasons. First, *CG31909* has a recently evolved *D. melanogaster*-specific near duplicate in that is also testes-expressed according to modENCODE and EST data (BT023668), and recently annotated as a protein-coding gene, *CG43800* (as of flybase release 5.45). This duplicate's function may be redundant with *CG31909* and sufficient to complement our TILLing mutant. Second, *CG31909* may be expressed in the testes but not essential for male fertility. Third, given that knockdown of *CG31909* by the Notch pathway promoter of *pannier* resulted in a lethal phenotype similar to other *de novo* genes, yet our nonsense and missense mutations had no effect on viability, *CG31909* may function in *viability* as a long non-coding RNA gene, despite the fact that it produces a protein.

### *De novo* genes in *D. melanogaster* are evolving rapidly

**Figure 7:**
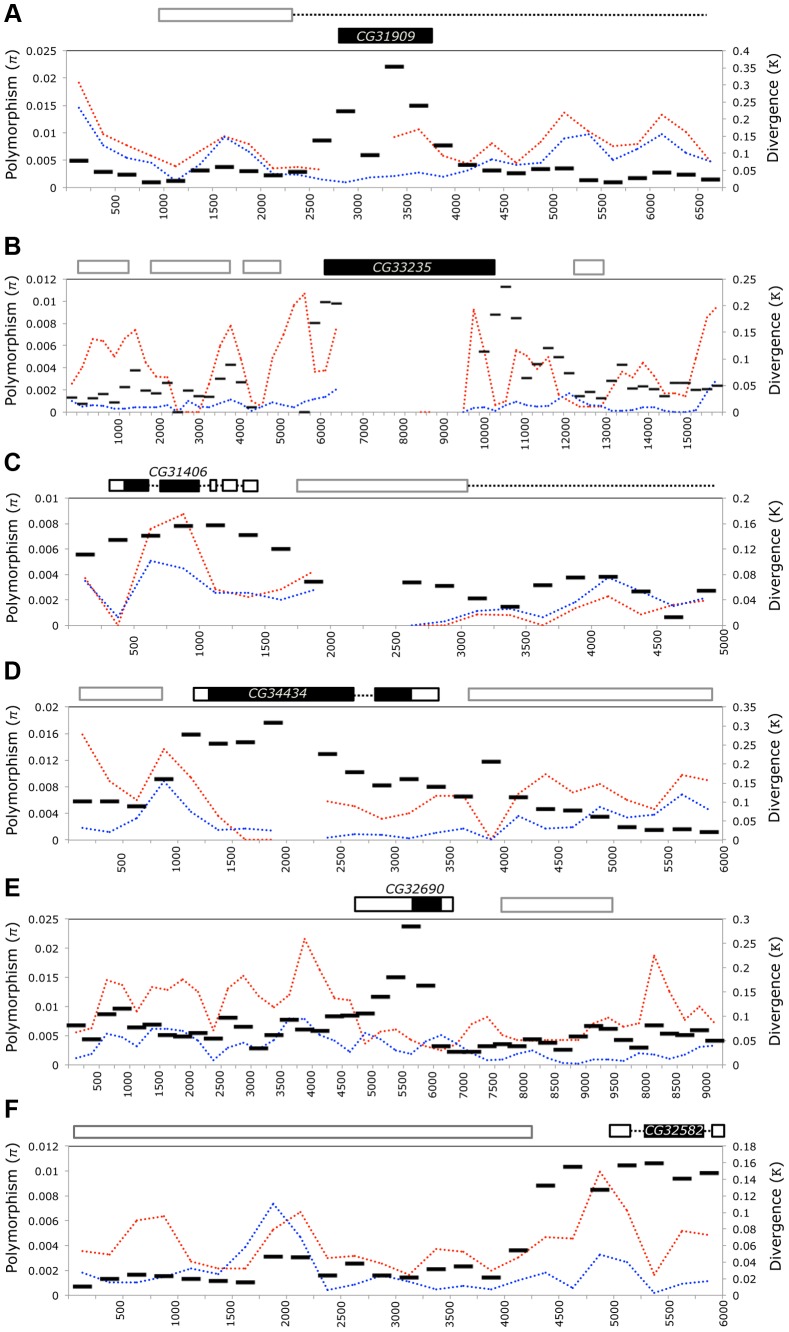
*D. melanogaster de novo* genes are highly diverged relative to neighboring sequences but carry little standing variation. The flanking gene region (5–15 kb) surrounding each *de novo* gene - *CG31909* (A), *CG33235* (B), *CG31406* (C), *CG34434* (D) *CG32690* (E), and *CG32582* (F) - was aligned to the collinear sequence from *D. simulans* (using MAUVE) and to *D. melanogaster* genomes from the Drosophila Population Genomics Project (www.dpgp.org). The length of the region used varies due to differences in colinearity with *D. simulans* (for example the area 3′ of *CG32582* is not present in *D. simulans*). We used Variscan to calculate pairwise divergence to *D. simulans* (*κ*, black bars) as well as polymorphism (*π*) from both the North American (blue) and African (red) populations. The large black block shows the position of the focal *de novo* gene, and surrounding outlined boxes are other genes in the region. Dashed lines indicate introns. Overall, the *de novo* genes show elevated divergence (but not polymorphism) relative to surrounding sequences, indicating they may have evolved through repeated selective sweeps, or that they evolved rapidly, and are now under purifying selection.

The *de novo* genes in our analysis are identified in part as being lineage-specific by a lack of sequence similarity to protein-coding genes in other species. Thus, it is unsurprising that these genes are highly diverged at the sequence level when compared to those relatives harboring orthologous sequence (Figure 2, Dataset S1). However, as we found many of these genes have become involved in essential functions, we expect that they have experienced strong selection as they acquire these functions. Where possible, we aligned the *D. simulans* and *D. melanogaster* extended gene region and compared with polymorphism data from *D. melanogaster* [34] (lines collected from Raleigh, USA, “NA” and Malawi, Africa “AF” as part of the DPGP project) using Variscan [35]. Divergence (Figure 7, *κ*, black bars) was always highest over the part of the region including the gene, whereas polymorphism was usually lower or similar to background levels (Figure 7, π, dotted lines). Furthermore, regions overlapping the CDS of *CG32582* and of *CG32690* had elevated rates of divergence compared to the entire transcribed region. An increased rate of divergence without a similar increase in polymorphism is generally consistent with positive selection acting on a gene. However, polymorphism-based metrics (Tajima's *D* and Fu and Li's *D* and *F* [36], [37]) failed to show significant deviation from neutrality for blocks containing the *de novo* genes (Table S3). Failure to reject the null could be due to low levels of polymorphism present within the open reading frames of the *de novo* genes and the small size of the genes combining to reduce the power of the test.

We also tested whether protein-coding regions of four genes with *D. simulans* ORFs (*CG34434*, *CG33235*, *CG31909* and *CG31406*) show signs of recent positive selection. Each gene had high levels of both synonymous and nonsynonymous divergence when compared to *D. simulans* (Table 2), but d_N_/d_S_ was below 1 in all cases, implying the genes are selectively constrained. None of the proteins tested show strong evidence that they have recently evolved under positive selection, though they are diverging rapidly at the sequence level. The DoS estimates and d_N_/d_S_ indicate that *CG31909* is the most likely of the four to be evolving under positive selection, though the McDonald-Kreitman test was not significant. On the other hand *CG33235* and *CG34434* show evidence of purifying selection (DoS is negative and d_N_/d_S_ are <1), despite high levels of nonsynonymous divergence. This makes sense given the evidence that these genes are essential for viability in *D. melanogaster*. For our six candidate *de novo* genes, the DPGP data show no evidence that any variants that disrupt the open reading frame are segregating (in the DPGP data set ∼3% of all genes harbor a segregating null [38]). In the case of *CG31909*, the region overlapping the gene was not found in the DPGP dataset, but a broad (300 allele) PCR-based survey of a natural population of *D. melanogaster* for deletions of *CG31909* found that in all cases, the gene was intact. Combined with our RNAi data the absence of common null mutations reinforces our observation that *de novo* genes have become important to fitness.

**Table 2:** Neutrality index and direction of selection estimates for four *de novo* genes.

	d_N_/d_S_	Dn	Pn	Ds	Ps	NI (Pn/Ps)/(Dn/Ds)	α	DoS Dn/(Dn+Ds)−Pn/(Pn+Ps)	MK test (G)	MK test P-value
CG33235	0.558	375	13	411	8	1.781	−0.781	−0.142	1.66	0.198
CG31406	0.605	52	8	35	7	0.769	0.231	0.064	0.217	0.641
CG31909	0.968	37	3	28	6	0.378	0.622	0.236	1.783	0.182
CG34434	0.342	103	16	68	7	1.509	−0.509	−0.093	0.765	0.382

## Discussion

Of the five *D. melanogaster de novo* genes we investigated in an RNAi screen, four RNAi lines resulted in lethality in our assay, three led to skewed sex-ratios in adults most likely due to sex-differential survival, and one showed altered male reproductive fitness (though this case may be a side effect of the reduced male viability in the same cross, Figure S2A). In short, *de novo* genes are consistently evolutionarily and biologically essential. In contrast, the origins of these genes are divergent—some *de novo* genes clearly began as (*de novo*) long RNAs, whereas others may have emerged from a proto-ORF, although it is clear that a proto-ORF is not required for their evolution. After they arose, *de novo* genes' sequence and structure invariably evolved rapidly. However, we did not detect significant signatures of recent positive selection, but this may be due to problems with power in the data (particularly the low levels of polymorphism). Earlier work suggested positive selection had acted on some of these genes [4].

RNAi knockdown caused lethality in four of five *de novo* genes tested, a surprising finding because these genes are very young—if these genes are essential, what function are they performing now that was apparently not needed by the ancestor? The lethality consistently occurred during late pharate adult stages (pre-eclosed adults), after full eye pigmentation and the appearance of bristles had begun (Figure 5). Expression of all the genes studied was high in both larvae and male adults, and this data suggests that the essential function of these genes begins prior to the adult stage. This implies that *de novo* genes are playing an important role in the development of the adult fly. Alternatively, during the sensitive pupation stage, the fly may not tolerate absence of a *de novo* gene even though this could be tolerated during larval development.

RNAi can have off-target effects, but we did not find evidence of knockdown of any genes predicted to be off targets by sequence similarity or lethality in genetic controls (Figure S1). Other large RNAi screens using similarly generated lines suggest that such off target effects are rare [17], [18] and that phenotypic effects produced by these lines are often confirmed with genetic mutants. It is impossible to completely rule out effects of RNAi on off-targets that have, for example, very weak sequence similarity to the double-stranded RNA, so extending this work using genetic mutants is a logical next step.

These strong effects on viability may appear at first to be at odds with the finding that expression of these genes is often strongest in the testes (Figures 3 and 4). Contrary to our naïve expectation, only one of the RNAi lines produced a defect in fertility (Figure 6, Figure S2B) and we interpret this effect to be a result of reduced robustness in RNAi males (Figure S2A). This pattern may be explained by global gene expression patterns. While nearly 20% of Drosophila genes show male-biased expression – a huge excess compared to other tissues [25], genes expressed in male germline stem cells prior to meiosis are typically also expressed in at least one other cell type [39]. Therefore, strong expression of a gene in the testes may not be a good indicator that a gene's function is testes or even male specific. For instance, we found that *CG31406* was under the regulation of a meiotic arrest gene, *tombola*, which functions in sperm development (Figure 3). Yet this gene had a strong effect on viability. Examples like this suggest that genes may be expressed at a high level due to general transcriptional “permissiveness” in the testes [40], [41], but their expression may not be critical to male reproduction. Alternatively, the strong testes expression may reflect the evolutionary origins of these genes rather their current function in the fly – that is, expression patterns may be conserved through phylogenetic momentum. This would be consistent with the hypothesis that the testes act as an “evolutionary playground” for the emergence of new genes that are later adapted to other functions [28].

Researchers have speculated that *de novo* genes may function as non-coding RNAs [13], [42], as seminal peptides (particularly in Drosophila, where they are often found to show expression in the male reproductive tract [4]–[6]), or may not be functional at all, but expressed as a side effect of nearby transcription or overly promiscuous transcription in particular tissues [28]. However, increasing evidence suggests that new genes of all forms, including *de novo* genes, are important to fitness. Our data suggest that in the time since these *de novo* genes arose they have integrated into some key developmental or physiological network and become critical to some basic function of the fly. These results parallel data from yeast [9], [16], which found that loss of a *de novo* gene in a synthetic lethal screen was lethal, and similar to work by Chen and colleagues showing that many types of young genes in Drosophila are essential [17]. Interestingly, although we tested only a handful of genes, this 80% “essentialness rate” is actually significantly (*P* = 0.035) higher than the ∼30% lethality rate observed for all classes of young genes *and* the 35% observed for old genes by Chen and colleagues. Thus, when a *de novo* gene arises and persists it appears even *more* likely than most other young genes to be integrated into an essential aspect of fly biology.

While our sample size is small and should be interpreted with caution, it is remarkable that so many of these genes appear to be essential. How can we explain this finding? The appearance of a wholly new gene would seem more likely than other types of mutation to result in a large phenotypic change. Models of both phenotypic and genotypic evolution predict that larger than expected changes occur early during a bout of adaptive evolution [43], [44]. While this may explain why the phenotypic effects of a new gene should be large it does not explain why these genes would become *essential* at a disproportionate rate. To become a gene that codes for a protein whose loss results in death, a *de novo* gene must become integrated into an essential physiological or developmental pathway. Unlike new duplicates - which often retain interacting partners with their parent genes - these genes are entirely novel and any interactions they have with other genes would be novel. Perhaps as the network adapts to the presence of a new member, the *de novo* gene becomes essential to network function and unlike new duplicates, if lost, interactions cannot be replaced by a parent copy. Interestingly, all of these proteins do have predicted interactions on the DroID database [45], including a substantial number of interactions with small RNAs. *CG31909*, for instance, is annotated as having interactions with six miR, including those important for development and ecdysone signaling (miR-125).

Our data show that two *de novo* genes first arose as non-coding RNAs. Although their ORFs are disrupted in non-*D. melanogaster* species, *CG32690* and *CG32582* are transcribed with a similar expression pattern across species. This pattern is similar to that seen in the mouse *de novo* gene, *Pldi*. Heinen and colleagues [13] argued that it is unlikely that a protein arising from a novel RNA would be functional and annotated their newly evolved transcripts as non-coding RNAs despite the presence of short open reading frames in these genes. However, our data suggest that for the other four genes considered in this study, the open reading frame may have been present when transcription began. Proteomic data from the EBI PRIDE database [21]–[24] showed evidence these “proto-ORF” *de novo* genes we identified do produce peptides in *D. melanogaster* (Table S2). Thus it seems unlikely that *de novo* genes function solely as RNA genes/lncRNAs, although we cannot reject the hypothesis that these protein coding *de novo* genes began as functional lncRNAs that later evolved an ORF, or that they may produce non-functional peptides and function primarily as lncRNAs.

Recent data suggests that a substantial fraction of non-coding DNA is experiencing natural selection [46]. Much of this selection is thought to be acting on regulatory sequences such as promoters and enhancers, and these types of changes are thought to be essential in adapting existing genes to perform new functions [47]. Our data suggests that selection is also shaping non-coding regions into functional protein coding genes are recruited into the basic and fundamental genetic pathways of the fly.

## Methods

### Molecular evolutionary annotation

Using data from Levine et al [4] and Zhou et al [20], we chose a number of published *de novo* genes to further characterize. In short, we combined the candidate genes from these two studies with an additional analysis comparing CDS of annotated *D. melanogaster* protein coding genes from FLYBASE (v4.3), which included a handful of partially annotated non-coding RNA genes, to the genomes of all other Drosophila species available at that time (tBLAST). Proteins that failed to have similarity to the any genomes outside the melanogaster clade we considered candidates. These candidates were then filtered (described below) and candidates were ruled in or out as *de novo* genes using currently existing data (Table S1). For example, the CDS of the genes presented have no significant hits by translated BLAST (e = 10∧−6) to genes outside of *D. yakuba/D. erecta*. We mined the NCBI trace archive to rule out the possibility that assembly error in species other than *D. melanogaster* had led to the misannotation of these genes as *de novo* and found no evidence these genes existed among the traces in species outside of what was previously reported. We searched UCSC's whole genome chained BLASTZ alignments, which are more sensitive to highly diverged hits than BLAST or BLAT [48] in order to find genomic regions collinear to the immediate gene regions in other species. We then used the UCSC [49] and Flybase [50] genome browsers to ask whether the *D. annanassae*, *D. yakuba*, *D. erecta*, *D. simulans*, and *D. sechellia* chained alignments covered annotated genes in whole or in part, despite not matching by BLAST/BLAT. Genes that were found to be collinear to annotated genes with similar structure in all five species were excluded as putative rapidly evolving loci (Table S1). In cases where gene structures were radically different, but there was overlap with an annotated gene, we used RT-PCR to verify (or exclude) the annotated gene models. In the case of *CG34434*, we found that the annotation of the putative *D. yakuba* ortholog incorrectly connected the putative ortholog of *CG34434* with a neighboring gene, and that the *D. simulans* gene had a second, unannotated exon similar to the second exon of the *D. sechellia* ortholog. These corrected gene structures were used in the presented analysis. Finally, the flybase annotation of the collinear *D. sechellia CG34434* ortholog (*GM12640*) had an incorrect splicing pattern leading to a frame-shifted second exon. Once corrected, *GM12640* was similar in sequence and structure to *CG34434*. We have contacted flybase and provided them with evidence for these updated annotations.

### Molecular evolutionary and population genetic analyses

We downloaded BLASTZ [48] alignments of the extended gene regions surrounding the six candidate *de novo* genes from the UCSC genome database. We used these alignments to determine which parts of the *D. melanogaster* putative lineage-specific genes and their flanking sequences were collinear to sequences in each of the other species. We extracted any portion of the alignment overlapping transcripts and realigned pairs of sequences (*D. melanogaster* against each other species) using the “water” pairwise alignment program, part of the EMBOSS suite [51]. We calculated the total sequence similarity and the proportion of alignable bases between sections of each gene (e.g. CDS, UTRs, etc) from these pairwise alignments.

We also performed a global pairwise alignment of the *D. melanogaster* and *D. simulans* extended gene regions (extracted from FlyBase genbank files) using progressiveMAUVE [52], [53]. We counted the number of fixed differences between *D. melanogaster* and *D. simulans* in 500 bp windows along the alignment, then aligned 39 *D. melanogaster* Raleigh genomes and 6–9 African genomes (www.dpgp.org, [34]) to these regions and calculated polymorphism (*π*) and divergence (*κ*) in each window. We looked for evidence of null alleles (e.g. premature stop codons in the DPGP data) and calculated Tajima's *D* [36] and Fu and Li's *D* and *F* [37] for 500 base pair windows across the region using Variscan [35]. For genes with intact proteins in *D. simulans*, we aligned the protein-coding regions using ClustalW and used these alignments to calculate the Neutrality Index (NI) and the Direction of Selection (DoS, [54]), and to perform a Macdonald-Kreitman test [55]. SNAP [56] was used to calculate d_N_/d_S_ relative to *D. simulans*, except in the case of *CG33235* where the comparison was to *D. sechellia* as that species has a longer ortholog than *D. simulans*.

Finally, in the case *CG31909*, data from DPGP was not available for most of the gene's CDS. Instead, we screened 150 wild caught African flies for deletions of *CG31909*, which would be expected to occur if the gene were non-essential. PCR was performed using primers (CTTGGCCCTGCGAAGTGAACACC and CGCACTGGGCGCTGAAATCTGTG) amplifying a ∼1 kb region surrounding *CG31909* looking for a negative reaction or short product. Candidates were then sequenced to confirm or deny the null allele.

### Tissue collection and dissection and expression analyses

Male reproductive tracts were dissected on ice from whole flies (*D. yakuba, D. simulans, and D. melanogaster*) in sterile PBS. Male reproductive tracts and carcasses were each pooled from at least 10 individuals and then flash frozen in liquid nitrogen. Whole females and males of each species were also collected, pooled and flash-frozen. *D. melanogaster*, *D. simulans*, and *D. yakuba* male reproductive tracts were further dissected into accessory glands and testes in PBS and flash frozen. *D. melanogaster* third instar larvae were sexed by identification of male and female genital discs following *Drosophila protocols* [26], then flash-frozen. Testes were dissected from males carrying a null mutation at the gene *tombola* (*tomb^GS12862^*, stock generously supplied by Dr. Helen White-Cooper), and sons of females mutant for the *tudor* gene (Bloomington stock #1786 – sons of these flies lack a male germline).

We extracted RNA from two or more biological replicates of each dissected tissue using TRIZOL reagent (Invitrogen, Grand Island, NY #15596-026), and synthesized cDNA using M-MLV reverse transcriptase (Invitrogen, Grand Island, NY #28025013). We performed relative qRT-PCR quantification using gene-specific primers and a single control primer that worked across all species (*Actin5c*). All qRT-PCR Ct values were averaged across two technical replicates.

In addition to our own data, we mined expression information from online databases - FlyAtlas [57], modENCODE RNAseq data [25], Baylor RNAseq data [58], and FlyTED: Testes expression database [59], and DroID [45]. Additionally, we mined Drosophila proteomic data from multiple sources [21]–[24]. These datasets are biased towards proteins expressed in early embryos as this constitutes ∼35% of available proteomic data and the handful of studies of testes and seminal fluid were of comparatively low depth [60]–[62].

### RNAi knockdown

Virgin *Actin5C*-GAL4 females (y1 w*; P{Act5C-GAL4}25FO1/CyO, y+, Bloomington 4414) were collected and crossed at 25C to lines carrying UAS-RNAi constructs for *CG33235*, *CG31909*, *CG31406*, *CG34434*, *CG32582* and *Gr22c* - a control obtained from VDRC [29] (stocks used: 19355, 23550, 39194, 41772, 102263, 104704, 105072, and 110307, 105051). *CyO* (control) and straight winged (RNAi) progeny of both sexes were counted and collected. For RNAi knockdown in larvae, we crossed the same RNAi lines to a stock with *Actin*-GAL4 and *CD8*::UAS-GFP on the same chromosome (y^1^ w*; P{Act5C-GAL4}25FO1, UAS:CD8:GFP/CyO, y, donated by S. Chen, [17]). In these crosses, RNAi or control status can be ascertained at any stage (RNAi larvae/pupae/adults will express GFP). We collected, sorted, and sexed larvae in the wandering stage and compared expression of the target gene using RT-PCR.

### Viability assays

To estimate effects on adult viability, we counted the number of control (*CyO*) and RNAi (straight-winged) progeny eclosing from the RNAi cross (described above). To determine the stage at which lethality was occurring, we crossed the same RNAi lines to a GFP marked *Actin*-GAL4 line (see above). We collected larvae from the cross during the late third instar wandering stage, and sorted by GFP expression and sex [26]). We then allowed RNAi (GFP) and control (no GFP) to continue development, and counted the number that survived or died prior to pupation or prior to eclosion.

## Supporting Information

Dataset S1 Protein alignments of *de novo* genes show disruption of the ancestral ORF in *CG32690* and *CG32582*.
(TXT)

Figure S1 RNAi knockdown of target and putative off-target genes. (A) We measured RNAi knockdown by comparing target gene expression in F1 RNAi individuals (Red/Pink bars) by crossing UAS-RNAi lines to *Actin*-GAL4 driver lines - y^1^ w*; P{Act5C-GAL4}25FO1/CyO, y+ - for the GD crosses and y^1^ w*; P{Act5C-GAL4}25FO1, UAS:CD8:GFP/CyO, y - donated by S. Chen - for the KK crosses) to their control siblings (Blue/Light Blue bars). Expression was compared to the reference gene *Actin5C* across at least 2 biological replicates and is shown relative to the control in each case. In the case of the “GD” lines knockdown was measured in adults. Knockdown was confirmed for *CG31406*, *CG33235*, and *CG34434* RNAi flies but was not confirmed in the *CG31909* RNAi flies (* P<0.05, ‡ P<0.1, NS P>0.1). In the case of the “KK” lines, knockdown was measured at the wandering larval stage because RNAi flies did not survive to adulthood and was confirmed for three of the genes tested, and was marginally significant for *CG32582* (P = 0.057). (B) For the KK lines, the expression of putative off-targets (as reported by VDRC) was also compared to rule out effects on viability being due to reduction in expression of an essential off-target gene (for *CG33350*, P = 0.098, all others NS).
(TIF)

Figure S2 *CG34434* RNAi flies have reduced lifespan and weak performance in a sperm competition assay. (A) *CG34434*-RNAi and control flies were sorted by sex and kept in small vial populations (5–10 flies) as they emerged, and were monitored for survival each day until all of the flies died. Flies of both sexes were flipped onto fresh food every 5 days and watered daily. RNAi males (light blue) died much more quickly than their female RNAi siblings (pink) or either control males or females (red, blue). (B) We used a sperm exhaustion assay to measure fertility in GD-RNAi flies for two *de novo* genes (*CG34434* and *CG33235*), and also compared the performance of males carrying two genetic mutations in the *de novo* gene *CG31909* (a null mutation R89* and a point mutation D118N) to a control cross using *w^1118^*. *CG34434* RNAi males (but no other genotype) showed a reduction in performance in this assay, with the effect becoming stronger over the 5 day mating period.
(TIF)

Table S1 Information about candidate *de novo* genes including rejected candidates.
(XLSX)

Table S2 Evidence of peptide expression for four of six *de novo* genes.
(XLSX)

Table S3 Nucleotide-based metrics of neutrality for *de novo* genes and surrounding regions and genes.
(XLSX)
