# Supplementary figures and images for: De Novo ORFs in Drosophila Are Important to Organismal Fitness and Evolved Rapidly from Previously Non-coding Sequences
Source: PLoS Genet. 2013 Oct 17;9(10):e1003860. doi: 10.1371/journal.pgen.1003860 (PMC3798262; doi:10.1371/journal.pgen.1003860)

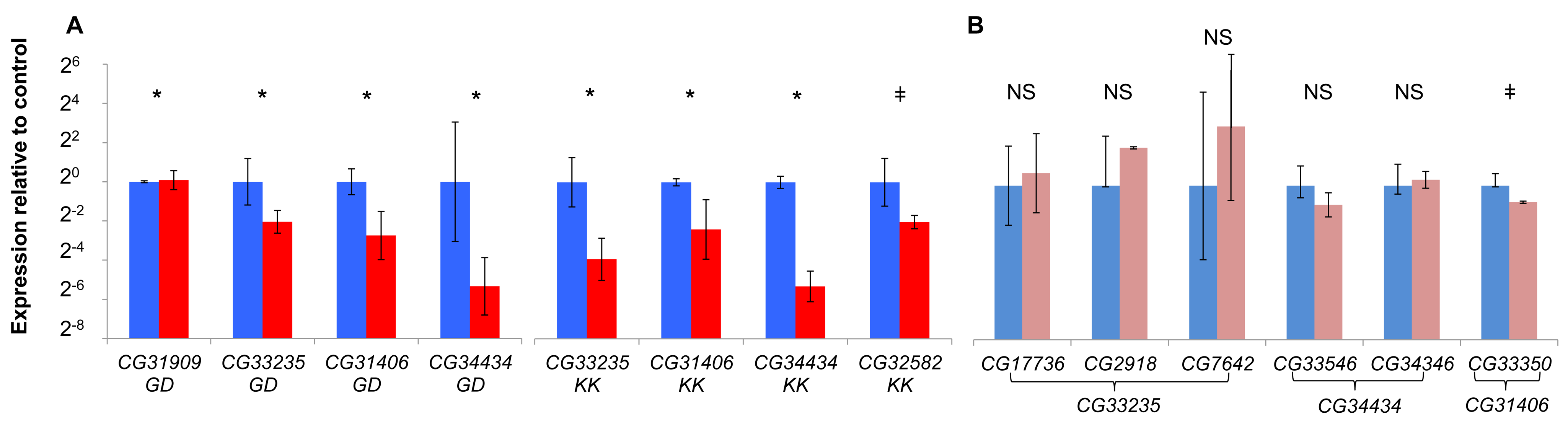

Supplement: Figure S1 — RNAi knockdown of target and putative off-target genes. (A) We measured RNAi knockdown by comparing target gene expression in F1 RNAi individuals (Red/Pink bars) by crossing UAS-RNAi lines to Actin-GAL4 driver lines - y1 w*; P{Act5C-GAL4}25FO1/CyO, y+ - for the GD crosses and y1 w*; P{Act5C-GAL4}25FO1, UAS:CD8:GFP/CyO, y - donated by S. Chen - for the KK crosses) to their control siblings (Blue/Light Blue bars). Expression was compared to the reference gene Actin5C across at least 2 biological replicates and is shown relative to the control in each case. In the case of the “GD” lines knockdown was measured in adults. Knockdown was confirmed for CG31406, CG33235, and CG34434 RNAi flies but was not confirmed in the CG31909 RNAi flies (* P<0.05, ‡ P<0.1, NS P>0.1). In the case of the “KK” lines, knockdown was measured at the wandering larval stage because RNAi flies did not survive to adulthood and was confirmed for three of the genes tested, and was marginally significant for CG32582 (P = 0.057). (B) For the KK lines, the expression of putative off-targets (as reported by VDRC) was also compared to rule out effects on viability being due to reduction in expression of an essential off-target gene (for CG33350, P = 0.098, all others NS). (TIF) [file pgen.1003860.s002.tif]

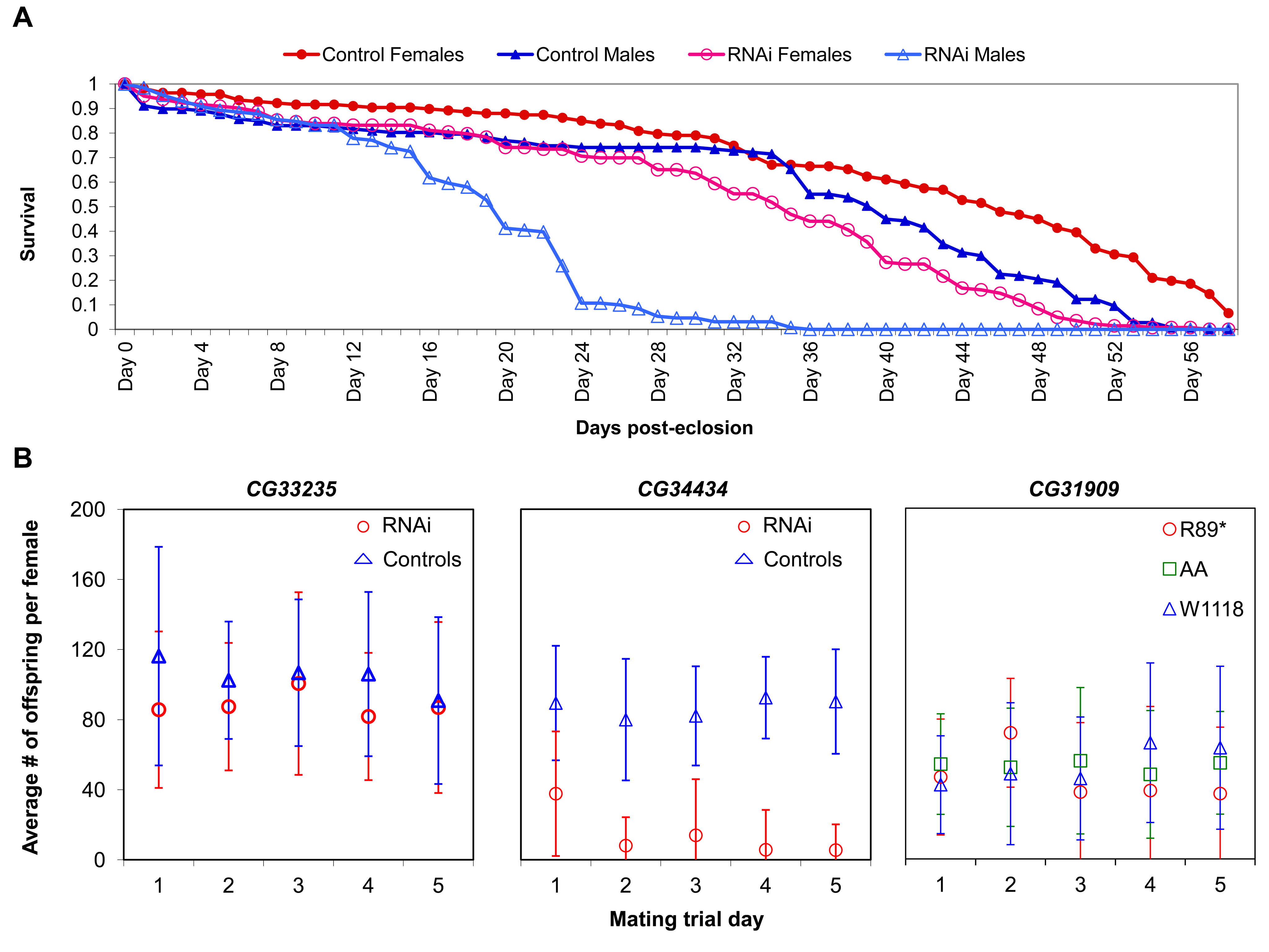

Supplement: Figure S2 — CG34434 RNAi flies have reduced lifespan and weak performance in a sperm competition assay. (A) CG34434-RNAi and control flies were sorted by sex and kept in small vial populations (5–10 flies) as they emerged, and were monitored for survival each day until all of the flies died. Flies of both sexes were flipped onto fresh food every 5 days and watered daily. RNAi males (light blue) died much more quickly than their female RNAi siblings (pink) or either control males or females (red, blue). (B) We used a sperm exhaustion assay to measure fertility in GD-RNAi flies for two de novo genes (CG34434 and CG33235), and also compared the performance of males carrying two genetic mutations in the de novo gene CG31909 (a null mutation R89* and a point mutation D118N) to a control cross using w1118. CG34434 RNAi males (but no other genotype) showed a reduction in performance in this assay, with the effect becoming stronger over the 5 day mating period. (TIF) [file pgen.1003860.s003.tif]
